# Roles of Endogenous Melatonin in Resistance to *Botrytis cinerea* Infection in an *Arabidopsis* Model

**DOI:** 10.3389/fpls.2021.683228

**Published:** 2021-06-21

**Authors:** Ying Zhu, Miao-Jie Guo, Jian-Bo Song, Shu-Yuan Zhang, Rui Guo, Dai-Ru Hou, Cheng-Ying Hao, Hong-Li An, Xuan Huang

**Affiliations:** ^1^Provincial Key Laboratory of Biotechnology of Shaanxi, Key Laboratory of Resource Biology and Biotechnology in Western China, Ministry of Education, College of Life Sciences, Northwest University, Xi’an, China; ^2^Center for Translational Medicine, The First Affiliated Hospital of Xi’an Jiaotong University, Xi’an, China

**Keywords:** melatonin, *Botrytis cinerea*, *Arabidopsis thaliana*, *N*-acetylserotonin methyltransferase, serotonin *N*-acetyltransferase

## Abstract

Melatonin is an important bioactive molecule in plants. Two synthetases, N-acetylserotonin methyltransferase (ASMT) and serotonin N-acetyltransferase (SNAT) are involved in the final two steps of melatonin synthesis. Melatonin participates in responses to a variety of biotic and abiotic stresses in plants, but few studies have addressed the roles of endogenous melatonin in pathogen resistance. We investigated the role of endogenous melatonin in resistance to *Botrytis cinerea* infection in an *Arabidopsis thaliana* model system. Plant lines that overexpressed *ASMT* or *SNAT* through genetic manipulation showed upregulated expression of resistance genes *PR1* and *PR5*, transcription factor gene *WRKY33*, and jasmonic acid (JA) defense pathway marker gene *PDF1.2*, and downregulated transcription factor gene *MYC2* in JA signaling pathway. Higher melatonin content also enhanced the activity of antioxidant enzymes superoxide dismutase (SOD) and peroxidase (POD), increased JA content, reduced plant disease symptoms, and reduced lesion size in leaves. These findings indicate that endogenous melatonin enhances plant resistance to *B. cinerea* infection. In contrast, *ASMT* and *SNAT* gene silencing lines showed opposite results and were more susceptible to *B. cinerea*. Thus, it can be demonstrated that melatonin functions as an effective regulator of plant stress resistance at the genetic level. A schematic model is presented for its role in resistance to *B. cinerea* infection. Our findings also helped to elucidate the associated signal transduction pathways and interactions between melatonin and other plant hormones.

## Introduction

Melatonin is a signaling molecule that ubiquitously exists in animal (Lerner et al., 1958) and plant (Dubbels et al., 1995) cells, and its synthesis pathways in plants have been discovered (Back et al., 2016). Serotonin *N*-acetyltransferase (SNAT; Kang et al., 2013) and *N*-acetylserotonin methyltransferase (ASMT; Park et al., 2013), as the main enzymes in the synthesis pathway, directly determine the endogenous melatonin level. The function of melatonin in plants has been widely reported (Fan et al., 2018; Sun et al., 2020). It can respond to various biotic (Mandal et al., 2018; Zhao et al., 2021) and abiotic (Chen et al., 2017; Yao et al., 2020) stresses to resist the influence of environmental changes during plant growth and development. The immune response of melatonin in plants requires the participation of many signaling molecules, such as reactive oxygen species (ROS; Pardo-Hernández et al., 2020) and Nitric Oxide (NO; Zhu et al., 2019), to transmit both intracellular and intercellular signals. In the field of biotic stress, melatonin is involved in plant resistance to numerous fungus (Moustafa-Farag and Almoneafy, 2019). The growth of certain plant fungi, including *Alternaria* spp. and *Fusarium* spp., is inhibited by treatment with melatonin. Increased melatonin levels in plants enhance resistance to *Sphaerotheca fuliginea* and oomycetes, and sensitivity to *Phytophthora infestans* (Zhang et al., 2017; Mandal et al., 2018). Melatonin pretreatment increases the resistance of apple (*Malus prunifolia*) to Marssonina apple blotch (*Diplocarpon mali*), by promoting expression of chitinase genes, regulating hydrogen peroxide (H_2_O_2_) and pathogenesis-related proteins (PR proteins; Yin et al., 2013). In banana (*Musa acuminata*), melatonin treatment induces the production of defense-related plant hormones [IAA, salicylic acid (SA), JA, ethylene] by regulating the expression of *MaHSP90*, thereby enhancing resistance to Fusarium wilt (Wei et al., 2017).

*Botrytis cinerea* often termed “gray mold,” is a necrotrophic pathogenic microorganism (fungus) responsible for major economic losses (related to its wide hosting range) considered second only to those of penicillin fungal pathogens (Dean et al., 2012). It kills host cells by secreting the sesquiterpene metabolite botrydial, and by producing ROS that induces oxidative outbreaks (Feng and Shan, 2014). *Botrytis cinerea* also degrades pectin in plant cell walls by synthesizing polygalacturonase, keratin enzyme, and cell wall degradation enzyme, thereby promoting invasion and damage to the plant (Liu et al., 2017). Local immune responses of plants to *B. cinerea* infection result in integration and expression of PR proteins, steady-state regulation of plant hormones, ROS production, and accumulation of secondary metabolites such as JA (Pieterse et al., 2009).

Plant hormone signal transduction is an important component of the local immune response, and JA in particular plays a crucial role in defense against *B. cinerea* (AbuQamar et al., 2017). In *Arabidopsis*, a JA-knockout mutant (*coi1*) defective in the perception of JA signal shows higher susceptibility to *B. cinerea* (Pingping et al., 2017). Transcriptional coactivator mediator subunit 25 (Med25) promotes plant resistance to *B. cinerea* by inducing the expression of the JA-dependent defensive gene (An and Mou, 2013). The invasion of *B. cinerea* to plants activates the expression of a marker gene of the JA pathway (plant defensin 1.2; *PDF1.2*; Frey et al., 2018). Conversely, *MYC2* plays a negative regulatory role in JA-mediated immunity and it interacts with JAZ [jasmonate zinc-finger inflorescence meristem (ZIM) domain] to promote JA decomposition, thereby reducing *MYC2* expression following *B. cinerea* infection (Liu et al., 2019). Transcriptome analyses indicate the alteration of thousands of transcripts in the body of an amoeba host following *B. cinerea* invasion, whereby mitogen-activated protein kinases (MAPKs) undergo phosphorylation changes during transcription of downstream genes, in which key transcription factors are involved in the regulation of plant defense responses (Mulema and Denby, 2012). WRKY proteins are major components of transcription factors that play essential roles in both PAMP triggered immunity (PTI) and Effector triggered immunity (ETI) during plant resistance to *B. cinerea* infection (Jones and Dangl, 2006). WRKY proteins regulate defensive responses to biotrophic and necrotized pathogens (Chen et al., 2018). WRKY33 also participates in the regulation of its expression through a clear feed-forward mechanism in combination with its promoter in *B. cinerea*-infected plants (Mao et al., 2011).

We investigated the role of endogenous melatonin in resistance to *B. cinerea* infection in an *Arabidopsis thaliana* model. Using genetic manipulation techniques, the expression of *SNAT* and *ASMT* was altered. After *B. cinerea* infection, we found that *SNAT* and *ASMT* overexpressed lines exhibit obvious disease resistance characteristics compared with Columbia (Col-0). On the contrary, disease-resistant ability in *SNAT* and *ASMT* mutant lines was weakened. The results clarified that melatonin can improve plant disease resistance to the stress of *B. cinerea* by regulating the expression of related genes and the content of phytohormone JA. These pieces of evidence further prove the role of melatonin in the field of biotic stress, especially for enhancing plant resistance to fungal invasion.

## Materials and Methods

### Plant Material and Pathogen Inoculation Procedure

*Arabidopsis thaliana* ecotype Col-0 was used in this study. Plants were grown for 4 weeks at 22°C under long-day conditions (16 h of light/8 h of dark) and light intensity 100 μmol·m^−2^·s^−1^.

The *B. cinerea* (B05.10) used in this study was cultivated on potato dextrose agar (PDA) medium (Zheng et al., 2015) in the dark for 5 days at a 28°C incubator to induce conidia production. Spores were collected and then suspended in 2% glucose solution to dilute to 1 × 10^5^ conidia/ml. Leaves (3–4 per plant) were added dropwise with 10 μl spore suspension, and kept in the dark for 24 h and then transfer to 16 h light/8 h dark condition and light intensity 100 μmol·m^−2^·s^−1^.

### Obtain Transgenic Plants

Using the technique of artificial miRNA (amiRNA; Schwab et al., 2006), we successfully constructed miR172-*snat* and miR172-*asmt* vectors. Then they were separately transferred into Col-0 to silence melatonin synthesis genes *via* the *Agrobacterium*-mediated transformation method. Seeds were selected by adding 50 mg/ml kan and 30 mg/ml cef in 1/2 MS medium and T3 generation transgenic lines (*asmt-1*, *asmt-2*, *snat-1*, and *snat-2*) were used in the following experiments. Genes overexpression lines (*ASMT-OE-1*, *ASMT-OE-2*, *SNAT-OE-1*, and *SNAT-OE-2*) were constructed in our laboratory by inserting *SNAT* and *ASMT* gene complementary DNA (cDNA) into the binary vector pRI101-*AN* (TaKaRa, Tokyo, Japan).

### Quantitative Real-Time RT-PCR Analysis

After inoculation with *B. cinerea* strains for 48 h, total RNA was isolated from leaves using RNeasy Plant Mini Kit (TaKaRa, Tokyo, Japan), then reverse-transcribed to cDNA with the PrimeScript™II1st strand cDNA Synthesis kit (TaKaRa, Tokyo, Japan). The expressions of relevant genes were evaluated by quantitative real-time RT-PCR (qRT-PCR; model CFX96; Bio-Rad).

The *Actin8* (AT1G49240) genes were used as internal controls. Each experiment was repeated at least three times.

### Determination of Activities of Antioxidant Enzymes Superoxide Dismutase and Peroxidase

After *B. cinerea* treatment for 24, 48, or 72 h, plant leaves (0.1 g) were ground separately in liquid nitrogen, incubated with 1.5 ml of phosphate buffer (PB; pH 7.8), and centrifuged (4,000 rpm, 15 min). The supernatant was taken as the solution to be tested.

Superoxide dismutase (SOD) and peroxidase (POD) activity was determined following Zang et al. (2015).

### Quantification of Melatonin and JA Content

Plant tissues (0.1 g) treated by *B. cinerea* for 24, 48, or 72 h were grounded in liquid nitrogen and added 900 μl 0.01 M PBS (pH 7.4), then ultrasonicated for 30 min and centrifuged (12,000 rpm, 10 min)at 4°C, The supernatants were ready to measure the content of melatonin and JA.

Melatonin content was quantified using the Melatonin Enzyme-Linked Immunosorbent Assay Kit (Jianglai, Shanghai, China), with absorbance read at 450 nm. JA content was determined using the Plant JA ELISA KIT (Jianglai, Shanghai, China) containing anti-JA polyclonal antibodies, with absorbance read at 490 nm. All measurements were performed in triplicate with samples collected from three biological replicates.

### Trypan Blue Staining

Leaves treated by *B. cinerea* after 3 days were stained with 0.4% (w/v) Trypan Blue solution for 2 h at 37°C, and chlorophyll was eliminated with 95% ethanol. The samples were observed under a dissecting microscope (Nikon C-LEDS; Nikon, Tokyo, Japan).

### Determination of Malondialdehyde Content

Following *B. cinerea* treatment for 24, 48, or 72 h, plant tissues (0.1 g) were ground in liquid nitrogen and added with 900 μl of PB. After centrifugation (4,000 rpm, 10 min), 50 μl of the supernatant was used as the reaction solution and assayed with an malondialdehyde (MDA) kit (Jiancheng, Nanjing, China). The absorbance of the supernatant was detected at the wavelengths of 532 nm.

### Leaf Damage Area Calculation

Leaves of plants treated with *B. cinerea* for 4 or 6 days were removed and photographed. Leaf damage area data were taken for the 6-day-treated plants and statistically analyzed using Image J 1.52 s software program (NIH Image, Bethesda, MD, United States).

### Statistical Analysis

Analyses were performed using the Origin software program V. 8.0, with values expressed as mean ± SE. Differences between means were evaluated by one-way ANOVA and considered significant for *p* < 0.05.

## Results

### Endogenous Melatonin Alters Stress Response in *B. cinerea*-Infected Plants

**Figure 1:**
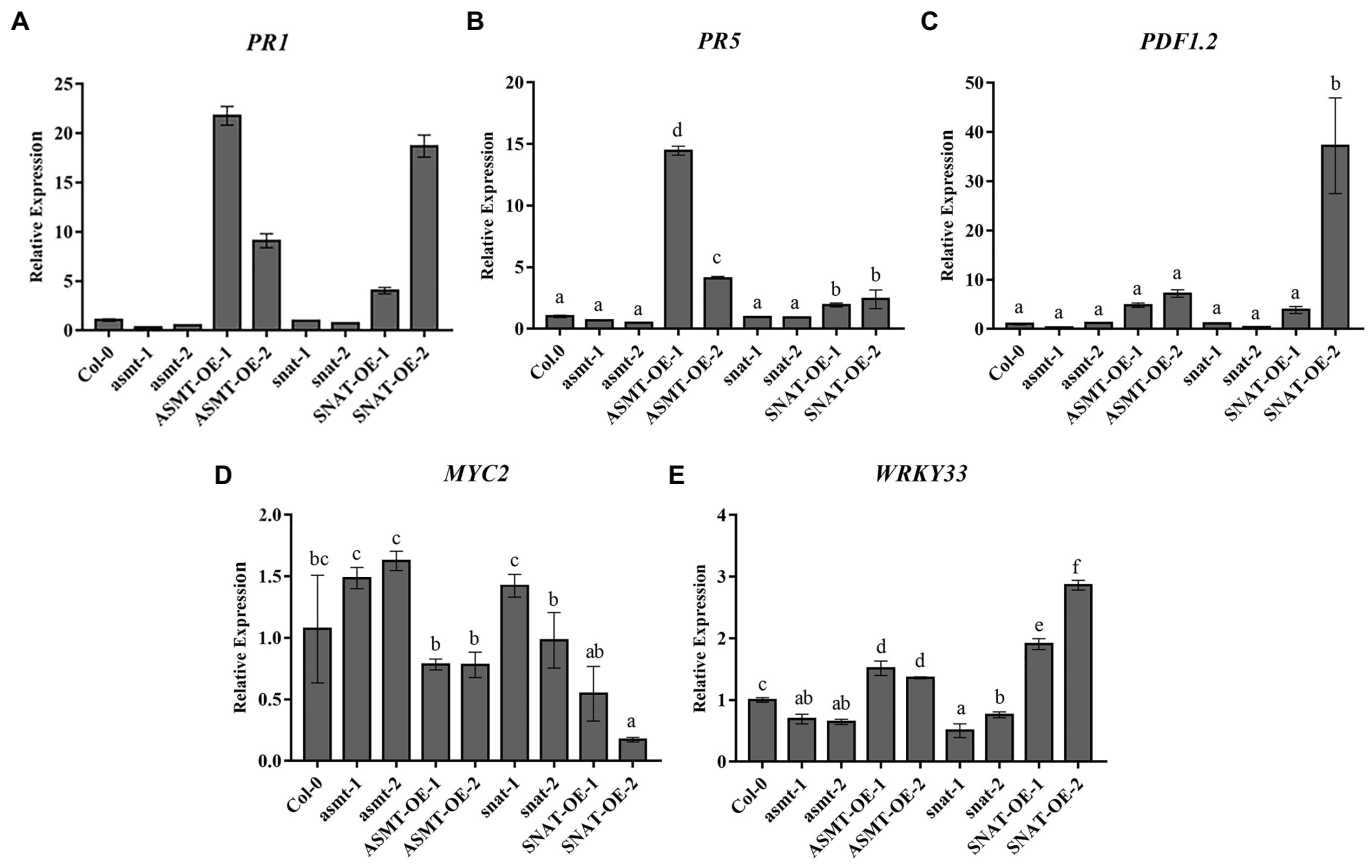
Expression of defends signaling-related gene following *Botrytis cinerea* treatment. The transcript levels of *PR1* **(A)**, *PR5* **(B)**, *PDF1.2* **(C)**, *MYC2* **(D)**, and **(E)** *WRKY33* were determined by RealTime Quantitative Polymerase Chain Reaction (RT-qPCR). RNA extracted from rosette leaves of 4-week old wild-type (Col-0), N-acetylserotonin methyltransferase (*ASMT*)-overexpressed (*ASMT-OE-1*, *ASMT-OE-2*) plants, serotonin N-acetyltransferase (*SNAT*)-overexpressed (*SNAT-OE-1*, *SNAT-OE-2*) plants, *snat* silencing lines (*snat-1*, *snat-2*), and *asmt* silencing lines (*asmt-1*, *asmt-2*) at 48 h after the *B. cinerea* infection. *PR1* (*AT2G14610*), pathogenesis-related protein 1; *PR5* (*AT1G75040*), pathogenesis-related protein 5; *PDF1.2* (*AT5G44420*), plant defensin 1.2; *MYC2* (*AT1G32640*); and *WRKY33* (*AT2G38470.1*). The data (mean ± SD) were calculated using three replicate assays, with the SEs indicated by the vertical bars. Different lowercase letters indicate statistically significant differences (*p* < 0.05).

To evaluate the role of endogenous melatonin in response to *B. cinerea* invasion and regulation of plant defensive signaling pathways, we analyzed the expression of resistance genes *PR1*, *PR5*, *PDF1.2*, *MYC2*, and *WRKY33* (Figure 1). Spore suspension droplets were taken from leaves of *B. cinerea*-infected plants at 48 h. *PR1* expression was significantly higher in the overexpression lines (most notably *ASMT-OE-1*) than in Col-0. In contrast, its expression was lower in the gene silencing lines (most notably *asmt-1*; Figure 1A). Expression of *PR5*, another important gene in defensive responses, was similarly increased in overexpression lines following *B. cinerea* infection (Figure 1B). Expression of *PDF1.2*, an important gene in the JA-dependent pathway, was increased 37-, 4.8-, 7-, and 2.8-fold (respectively) in *SNAT-OE-2*, *ASMT-OE-1*, *ASMT-OE-2*, and *SNAT-OE-1* relative to Col-0. In contrast, its expression was lower in *asmt-1* and *snat-2* (respectively ~33 and 37% of Col-0 value), and not notably different in *asmt-2* or *snat-1* (Figure 1C). Expression of *MYC2*, a negative regulatory gene in the JA-dependent signaling pathway, showed an opposite trend. It was lower in overexpression lines (*SNAT-OE-2* value ~17% of Col-0 value) and increased in gene silencing lines (Figure 1D). Expression of *WRKY33*, a transcriptional regulator gene, varied less in the modified lines than that of *PR1* or *PR5*. The transcription level of *WRKY33* was increased in the *SNAT*-overexpression lines, particularly *SNAT-OE-2*, whose value was 2.86 times that of Col-0. *WRKY33* expression was lower in gene silencing lines, most notably *snat-1* (value ~50% that of Col-0; Figure 1E).

These findings indicate that the expression of *ASMT* and *SNAT* genes plays an important role in regulating the expression of *PR1*, *PR5*, *PDF1.2*, *MYC2*, and *WRKY33*. Downregulation of *ASMT* and *SNAT* inhibits expression of *B. cinerea* infection resistance genes, thereby increasing susceptibility of plants to fungal pathogens. Upregulation of *ASMT* and *SNAT* in overexpression lines enhances resistance to *B. cinerea*. In summary, plant defensive responses to *B. cinerea* infection are strongly affected by alterations in levels of the two melatonin synthesis enzymes or of endogenous melatonin.

### Effects of Melatonin Levels on Resistance to *B. cinerea* Infection

**Figure 2:**
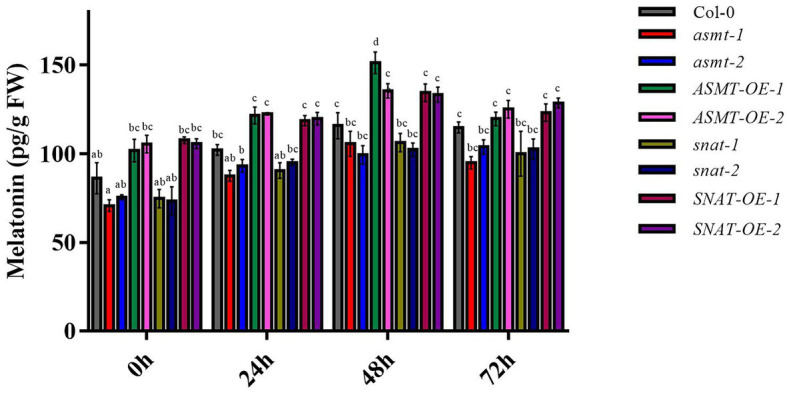
Melatonin content following *B. cinerea* treatment. The content of melatonin in 4-week old wild-type (Col-0), *ASMT*-overexpressed (*ASMT-OE-1*, *ASMT-OE-2*) plants, *SNAT*-overexpressed (*SNAT-OE-1*, *SNAT-OE-2*) plants, *snat* silencing lines (*snat-1*, *snat-2*), and *asmt* silencing lines (*asmt-1*, *asmt-2*) were measured at different time points (0, 24, 48, and 72 h) after inoculation with *B. cinerea*. The data (mean ± SD) were calculated using three replicate assays, with the SEs indicated by the vertical bars. Different lowercase letters indicate statistically significant differences (*p* < 0.05). FW, fresh weight.

To further investigate the roles of *ASMT* and *SNAT* genes in pathogen resistance, we measured endogenous melatonin levels in the overexpression and gene silencing lines. Melatonin levels were higher after 48 h *B. cinerea* treatment relative to 0 or 24 h treatment, most notably for *ASMT-OE-1* (Figure 2). The increase was significant for overexpression lines, but not for gene silencing lines or Col-0. Melatonin levels were lower in all lines at 72 h, most notably for *asmt-1*. These findings indicate that endogenous melatonin content is affected by up- or downregulation of *ASMT* and *SNAT* genes, suggesting a positive regulatory effect of endogenous melatonin in resistance to *B. cinerea* infection.

### Effects of Endogenous Melatonin on Pathogenic Processes in *B. cinerea*-Infected Plants

**Figure 3:**
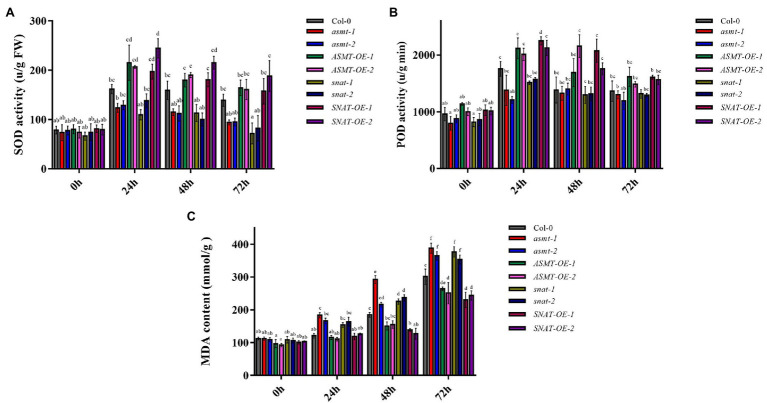
The activities of antioxidant enzymes **(A)** superoxide dismutase (SOD) activity and **(B)** peroxidase (POD) activity and **(C)** the content of malondialdehyde (MDA) following *B. cinerea* treatment. Significant differences among 4-week old wild-type (Col-0), *ASMT*-overexpressed (*ASMT-OE-1*, *ASMT-OE-2*) plants, *SNAT*-overexpressed (*SNAT-OE-1*, *SNAT-OE-2*) plants, *snat* silencing lines (*snat-1*, *snat-2*), and *asmt* silencing lines (*asmt-1*, *asmt-2*) at different time points (0, 24, 48, and 72 h) after the *B. cinerea* infection. The data (mean ± SD) were calculated using three replicate assays, with the SEs indicated by the vertical bars. Different lowercase letters indicate statistically significant differences (*p* < 0.05). FW, fresh weight.

Superoxide dismutase is resistant to oxidative damage, and plays a key role in the removal of free radicals in plants (López-Cruz et al., 2016). SOD activity in our plant lines varied depending on *B. cinerea* treatment time (Figure 3A). At 24 and 48 h, SOD activities of overexpression lines were higher than that of Col-0, whereas those of gene silencing lines were significantly lower. SOD activities of the various lines were generally lower at 72 h than at 48 h, but the overall trend was consistent. POD activities in the lines did not differ significantly at 0 h, similarly to SOD activities (Figure 3B). These findings indicate that *ASMT* and *SNAT* cope with peroxide damage by alterations of endogenous melatonin level, thereby enhancing plant resistance to fungal invasion.

Spores (conidia) of *B. cinerea* attach to the plant surface and germinate, and the degree of leaf damage is proportional to infection time (Williamson et al., 2007; Wang et al., 2013). *Botrytis cinerea* treatment did not initially cause notable damage to plants, and MDA content at 0 h did not differ significantly among the lines, similarly to SOD activity. At longer treatment times, *B. cinerea* spores reproduced parasitically on leaves, leading to a greater degree of lipid peroxidation and more damage to the plants. MDA content was the highest at 72 h in all strains (Figure 3C). These findings indicate that upregulation of *ASMT* and *SNAT* enhances endogenous melatonin level and reduces cell membrane damage, thus increasing resistance to *B. cinerea* and relieving biotic stress, whereas downregulation of *ASMT* and *SNAT* has the opposite effect.

### Effects of Leaf Damage in *B. cinerea*-Infected Plants

**Figure 4:**
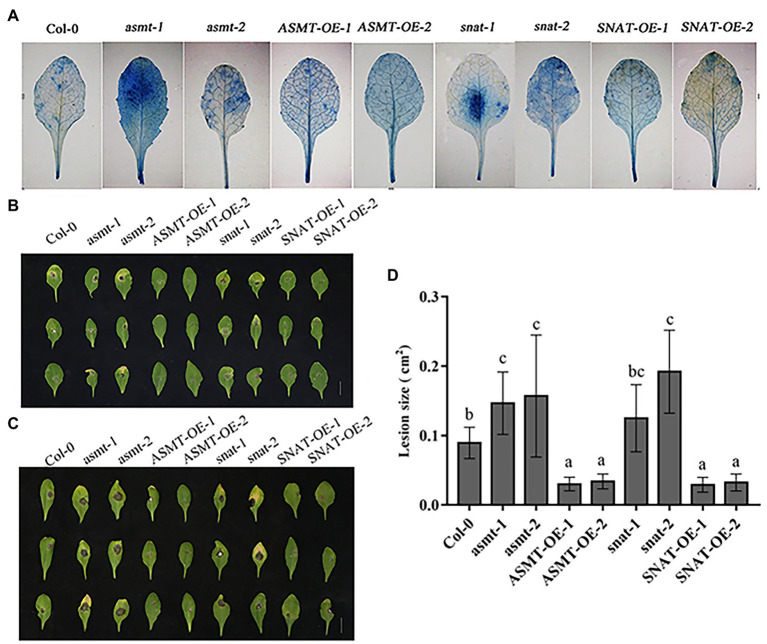
Effects of endogenous melatonin on disease resistance to *B. cinerea*. **(A)** Trypan blue staining of leaves from different lines following *B. cinerea* treatment at 48 h. **(B,C)** Photographs of rosette leaves cut from 4-week-old Wild-type (Col-0), *ASMT*-overexpressed (*ASMT-OE-1*, *ASMT-OE-2*) plants, *SNAT*-overexpressed (*SNAT-OE-1*, *SNAT-OE-2*) plants, *snat* silencing lines (*snat-1*, *snat-2*), and *asmt* silencing lines (*asmt-1*, *asmt-2*) plants after infection with *B. cinerea* spores. **(B)** Leaves phenotype on 4 days. **(C)** Leaves phenotype on 6 days. **(D)** Lesion size. Lesion size of leaves phenotype on 6 days was measured using Image J software. The data (mean ± SD) were calculated using three replicate assays, with the SEs indicated by the vertical bars. Different lowercase letters indicate statistically significant differences (*p* < 0.05).

*Botrytis cinerea* infection of leaves results in damage to cell membranes, such that the dye can enter cells. The degree to which cells are dyed blue reflects the extent of the disease (Ramírez et al., 2011). In Col-0, blue spots were scattered on various parts of the leaves. Blue spots on *asmt-1*, *snat-1*, *asmt-2*, and *snat-2* leaves were deeply colored, indicating presence of more dead cells and greater susceptibility to *B. cinerea*. The overexpression lines showed only a few scattered dark blue spots, indicating a lesser degree of leaf cell death than in Col-0, and greater resistance to *B. cinerea* invasion (Figure 4A).

Reactions to *B. cinerea* infection varied among Col-0, gene silencing lines, and overexpression lines. At 4 days after inoculation (dai), gene silenced lines than in Col-0 showed greater susceptibility to *B. cinerea* more severe disease symptoms and more fungal growth. In contrast, overexpression lines showed lesser and more slowly developing disease symptoms, small disease area, and limited spore growth (Figure 4B). Leaf necrosis was more severe on 6 dai (Figure 4C) than on 4 dai (Figure 4B) for gene silencing lines, but overexpression lines showed only a minor difference between these days. These findings indicate that *ASMT* and *SNAT* gene knockdown reduces plant resistance to *B. cinerea* infection.

A graph of lesion size in the various lines (Figure 4D) also illustrates the relationship between *B. cinerea* and *ASMT*/*SNAT*. Leaf damage area for the four-gene silencing lines (most notably *snat-2*) was significantly larger than for Col-0. Upregulation of *ASMT* and *SNAT* promoted host defensive response.

### Effect of Endogenous Melatonin on JA Content

**Figure 5:**
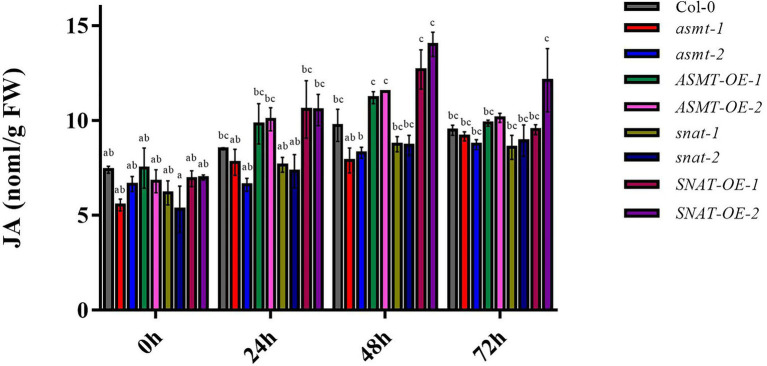
Jasmonic acid (JA) content following *B. cinerea* treatment. The content of JA in 4-week-old wild-type (Col-0), *ASMT*-overexpressed (*ASMT-OE-1*, *ASMT-OE-2*) plants, *SNAT*-overexpressed (*SNAT-OE-1*, *SNAT-OE-2*) plants, *snat* silencing lines (*snat-1*, *snat-2*), and *asmt* silencing lines (*asmt-1*, *asmt-2*) plants at different time points (0, 24, 48, and 72 h) after inoculation with *B. cinerea*. The data (mean ± SD) were calculated using three replicate assays, with the SEs indicated by the vertical bars. Different lowercase letters indicate statistically significant differences (*p* < 0.05). FW, fresh weight.

Jasmonic acid signaling plays a key role in the *B. cinerea* infection process. We examined the effects of altered endogenous melatonin levels on JA content. At 0 h, JA content did not differ notably among Col-0, gene silencing lines, and overexpression lines. JA content was significantly higher at 24 h than at 0 h for overexpression lines (particularly *SNAT-OE-1*), but such change was less notable for gene silencing lines (Figure 5). Maximal values were observed at 48 h for Col-0 and overexpression lines. JA content at 48 h was significantly higher for overexpression lines (particularly *SNAT-OE-2*) than for Col-0. Values at 72 h were lower than those at 48 h for Col-0 and all four overexpression lines. These findings indicate that endogenous melatonin level affects JA content for signal transduction pathways involved in pathogen resistance.

## Conclusion and Future Perspective

*Botrytis cinerea* is a necrotrophic fungus with a broad host plant spectrum (Dean et al., 2012), Plants are infected mainly by *B. cinerea* spores, which are released from previously infected plants when disturbed (Williamson et al., 2007). Some researchers have uncovered the role of melatonin in plant-*B. cinerea* interaction, for instance, tomato (Liu et al., 2019), *Fragaria ananassa* (Aghdam and Fard, 2017). We are going to study the resistance of melatonin to *B. cinerea* or related fungal species in *A. thaliana* and other model plants in future work. Enhancing effects of exogenous melatonin on plant resistance to fungi have been observed in many studies (Amaral and Cipolla-Neto, 2018). Much less is known regarding the role of melatonin (particularly endogenous melatonin) in plant resistance to *B. cinerea*. We examined the effects of altered endogenous melatonin levels on such resistance.

Plant defense mechanisms against pathogens involve complex signaling networks, including the expression of related genes (Senthil-Kumar and Mysore, 2013). *PR1* gene often plays a key role in plant resistance against necrotrophic pathogens (Frey et al., 2018). In this study, *PR1* expression in *ASMT* and *SNAT* overexpression lines was significantly increased relative to Col-0 but was reduced in gene silencing lines (Figure 1A). These findings are consistent with previous reports that the increase of *PR1* expression promotes plant defense against *B. cinerea* (Frey et al., 2018). *PR1* is considered to be associated with SA; however, it shows increased expression mainly in defensive responses against biotrophic fungi and not those against necrotrophic *B. cinerea*. Functional roles of SA are complex, and functions of SA signals in plant resistance to *B. cinerea* remain unclear (AbuQamar et al., 2017). *PR5*, a gene co-expressed with *PR1*, is also involved in signal transduction in plants (Siewers et al., 2005). We observed upregulation of *PR5* in overexpression lines, similarly to *PR1*, and downregulation in *asmt-1* and *asmt-2*. However, *PR5* expression in *snat-1* and *snat-2* did not differ notably from that in Col-0, possibly because *PR5* is not a single-label gene for resistance to *B. cinerea* (Figure 1B).

Transcription factor WRKY33 is an essential component in the regulation of plant defensive responses to fungal infection. *WRKY33* expression is induced in *B. cinerea*-infected plants. In our gene expression analysis, *WRKY33* was upregulated in overexpression lines but downregulated in gene silencing lines (Figure 1E), consistently with the increased susceptibility to *B. cinerea* observed for *WRKY33* mutants (*wrky33-1*, *wrky33-2*). In previous studies, *WRKY33* has been suggested to participate in JA-dependent pathways and to play a negative regulatory role in JA-mediated defensive responses. However, we did not observe an association between *WRKY33* increase and reduced JA content (Figure 5), indicating that this gene is not involved in the JA-dependent signaling pathway in this case. Recent reports show that *WRKY33* induction in *B. cinerea*-infected plants does not require a JA signal molecule (CORONATINE INSENSITIVE1; COI1; Wang et al., 2015), which illustrates that WRKY33 is activated *via* a JA-independent pathway. On the other hand, WRKY33 is phosphorylated by MPK3/MPK6 to induce synthesis of the phytoalexin camalexin in *B. cinerea* infected plants (Mao et al., 2011), suggesting that *WRKY33* expression in our *ASMT* and *SNAT* overexpression and gene silencing *Arabidopsis* lines may be related to MPK3/MPK6. We speculate melatonin can act directly on MAPKs and it further phosphorylates *WRKY33*.

Changes in growth and physiological processes of *B. cinerea*-infected plants are related to a variety of metabolic processes, including enzyme degradation and soluble sugar accumulation in cell walls, pH changes, and reduced production of antifungal compounds and secondary metabolites. Most of these changes are regulated by hormone signals, such as ethylene, Abscisic Acid, Jasmonic Acid and Salicylic Acid (Ingle et al., 2015). Studies based on transcriptome analysis suggest that the involvement of melatonin in plant-fungus interactions alters the expression of JA-related genes, and that melatonin can interact with JA to modulate plant defensive responses (Mandal et al., 2018). However, the role of melatonin in the JA pathway remains unclear. We observed high JA content (Figure 5) in our overexpression lines, but low JA content in gene silencing lines. Thus, increased melatonin level promoted JA accumulation, and consequently resistance to fungal infection. Increased JA content triggers signaling pathways downstream of JA. JAZ protein has an inhibitory effect on JA signaling pathway, and increased JA content leads to JAZ protein degradation, thereby reducing interaction between JAZ and MYC2 (Ruan et al., 2019), consistently with the reduced *MYC2* transcription levels in our overexpression lines (Figure 1D). MYC2 is a regulatory factor that plays an essential role in the JA-dependent signaling pathway. It negatively regulates downstream signal genes (*ORA59*/*ERF1*) that activate plant defensive responses and trigger downstream expression of *PDF1.2*, which plays a positive regulatory role in JA-dependent disease resistance (Frey et al., 2018). Consistently, *PDF1.2* expression was increased strongly in overexpression line *SNAT-OE-2*, and also in *ASMT-OE-1*, *ASMT-OE-2*, and *SNAT-OE-1*, but was reduced in gene silencing lines *asmt-1* and *snat-2* (Figure 1C). These findings further confirm the involvement of melatonin in JA signaling pathway activation and in the enhancement of plant resistance to *B. cinerea* infection. ROS are among the earliest signaling molecules in the interaction between plants and pathogens. Plants have evolved a variety of enzymes, and non-enzyme antioxidant defense systems, that promote the removal of ROS and prevent oxidative damage to plant tissues. SOD and POD are well-studied antioxidant enzymes, and have been shown to help regulate oxidative reaction balance in resistance to *B. cinerea*. In our study, SOD and POD activities did not vary notably among the various lines at 0 h but increased as *B. cinerea* treatment time increased (Figures 3A,B). Similar results were obtained in studies of two apple cultivars (Bui et al., 2019). In our study, MDA content in all lines reached maximal value at 72 h (Figure 3C), possibly because the reduction of SOD and POD activities did not allow prompt relief of oxidative stress resulting from ROS accumulation. MDA levels in Col-0 and our gene silencing lines and overexpression lines were similar at later treatment times, as well as at 0 h.

Increased ROS levels in response to external stimuli in plants led to a rise in melatonin levels and consequent activation of antioxidant enzyme activity (Liu et al., 2019). We observed changes in endogenous melatonin levels (Figure 2) resulting from up- or downregulation of *ASMT* and *SNAT* genes. Melatonin acts synergistically with antioxidant enzymes to remove ROS, promote photosynthesis, delay metabolite biosynthesis, enhance antioxidant capacity of plants, reduce oxidative stress in cells, tissues, or whole organisms, and protect plants from harsh environments (Manchester et al., 2015). Thus, increased endogenous melatonin promotes SOD and POD activity, facilitating prompt removal of excess ROS, and thereby enhancing plant defense against *B. cinerea*. Similarly, melatonin reduces damage to citrus fruits by penicillin fungal pathogens by removing ROS.

Various molecules secreted by *B. cinerea* induce the death of host cells (Siewers et al., 2005). Trypan blue staining revealed fewer dead cells in overexpression lines, and more dead cells in gene silencing lines, relative to Col-0 (Figure 4A). A possible explanation is that necrotrophic stenosis induces ROS accumulation, triggering programmed cell death (PCD) in host cells and providing nutrients to fungi, thereby promoting their growth and reproduction, and the appearance of disease symptoms (Torres, 2010). Infection of leaves by *B. cinerea* results in obvious necrotic symptoms (Liu et al., 2017). *Botrytis cinerea* infection of apple plants similarly caused significant disease symptoms (Bui et al., 2019). Phenotypic observations of leaves in the present study revealed disease symptoms on 4 dai, and the appearance of large necrotic lesions by 6 dai (Figures 4B,C). Overexpression lines were less susceptible to *B. cinerea* relative to Col-0, whereas gene silencing lines were more susceptible and developed more obvious lesions (Figure 4D). Our findings suggest that *ASMT* and *SNAT* overexpression enhances plant resistance to *B. cinerea* by increasing melatonin level, consistently with previous reports that plant resistance to other fungi is enhanced by endogenous melatonin.

The growth of fungi was inhibited in a PDA medium containing melatonin, suggesting that melatonin enhances plant resistance (Arnao and Hernández-Ruiz, 2015). At various times (24, 48, and 72 h) following *B. cinerea* inoculation, the induced substrate for melatonin synthesis increases, and *SNAT* and *ASMT* overexpression leads to further melatonin synthesis (Figure 2), suggesting that melatonin is involved in resistance to *B. cinerea*. Plant infection with the pathogen *Pseudomonas syringae* pv. tomato DC3000 (*Pst* DC3000) similarly caused the increase of melatonin levels (Shi et al., 2015). Melatonin levels were high in our overexpression lines but lower in gene silencing lines. Melatonin content was maximal in *ASMT-OE-1* at 48 h, in *ASMT-OE-2* at 24 h, and in *SNAT-OE-2* at 72 h. This finding suggests that melatonin is being synthesized *via* multiple biosynthetic pathways.

**Figure 6:**
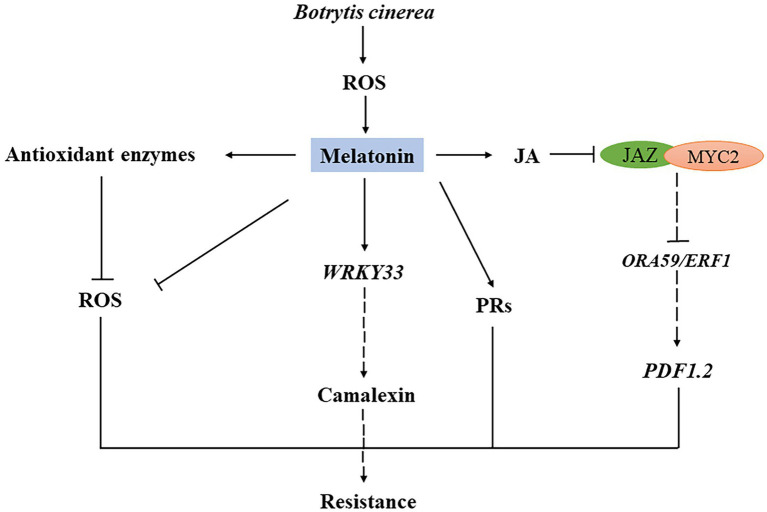
Schematic model of the role of melatonin in plant resistance to *B. cinerea* infection. Under *B. cinerea* Infection, endogenous melatonin content increases and decreased transcript levels of *JAZ1* and *MYC2*, which are two negative genes in the JA signaling pathway. Increasing Increased melatonin content also reduces ROS level by stimulating antioxidant system enzyme genes. The increase of melatonin content leads to the upregulation of *WRKY33* and defense genes (*PRs*, *PDF1.2*) expression.

In conclusion, we observed that increased endogenous melatonin level enhanced plant resistance to *B. cinerea*, consistently with previous reports. Melatonin is involved in the basic defensive responses of plants to *B. cinerea* and plays an essential role in plant immunity. Our findings provide new insights into molecular mechanisms of plant defensive signaling initiated by melatonin during interaction with pathogens, particularly fungal pathogens (Figure 6).

## Data Availability Statement

The original contributions presented in the study are included in the article/Supplementary Material, further inquiries can be directed to the corresponding authors.

## Author Contributions

The authors declare that the work described was original research that has not been published previously, and not under consideration for publication elsewhere, in whole or in part. All the authors listed have approved the manuscript that is enclosed. All authors contributed to the article and approved the submitted version.

### Conflict of Interest

The authors declare that the research was conducted in the absence of any commercial or financial relationships that could be construed as a potential conflict of interest.
