# Supplementary material for: Roles of Endogenous Melatonin in Resistance to Botrytis cinerea Infection in an Arabidopsis Model
Source: Front Plant Sci. 2021 Jun 21;12:683228. doi: 10.3389/fpls.2021.683228 (PMC8256269; doi:10.3389/fpls.2021.683228)
Supplement: Supplementary file 1 [file Data_Sheet_1.PDF]

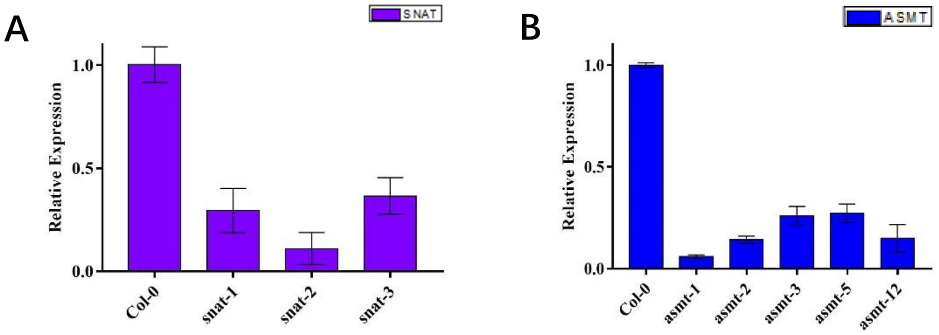

Figure.7. qRT-PCR results of *SNAT* and *ASMT* expression levels in silenced transgenic plants. A. The expression levels of *SNAT* in four-weeks old Wild-type (Col-0) and *SNAT* silencing lines. Compared with Col-0, *SNAT* in *snat-1* line decreased to 0.29, which was about 3.45 times lower. The *SNAT* of *snat-2* line decreased to about 0.11, which was about 9 times lower than that of Col-0. B. The expression levels of *ASMT* in four-weeks old Wild-type (Col-0) and *ASMT* silencing lines. The results showed that in the *ASMT* silenced transgenic plants, the expression levels of *ASMT* gene were significantly reduced in *asmt*-1 and *asmt-2* compared with Col-0. *ASMT* in *asmt-1* line decreased to 0.05, which was 20 times lower. The *ASMT* of *asmt-2* line decreased to about 0.14, which was about 7.14 times lower. The data (mean ± SD) were calculated using three replicate assays, with the standard errors indicated by the vertical bars.

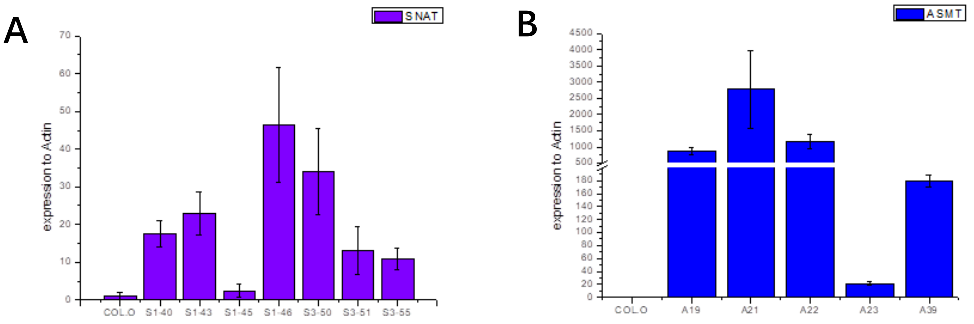

Figure.8. qRT-PCR results of *SNAT* and *ASMT* expression levels in overexpressed transgenic plants. A. The expression levels of *SNAT* in four-weeks old Wild-type (Col-0) and *SNAT* overexpressed lines. Compared with Col-0, *SNAT* in *SNAT-OE-46* plants line increased to about 47, which was about 47 times higher. The *SNAT* of *SNAT-OE-50* line increased to about 35, which was about 35 times higher than that of Col-0. B. The expression levels of *ASMT* in four-weeks old Wild-type (Col-0) and *ASMT* overexpressed lines. The results showed that in the *ASMT* overexpressed transgenic plants, the expression levels of *ASMT* gene were significantly increased in *ASMT-OE-21* and *ASMT-OE-22* compared with Col-0. *ASMT* in *ASMT-OE-21* line increased to about 2750, which was about 2750 times higher. The *ASMT* of *ASMT-OE-22* line increased to about 1250, which was about 1250 times higher. The data (mean ± SD)

were calculated using three replicate assays, with the standard errors indicated by the vertical bars.

In this experiment, lines with significantly different expression levels than Col-0 were selected. Lines of *snat-1*, *snat-2*, *asmt-1* and *asmt-2* were treated as silenced transgenic plants. Meanwhile, lines of *SANT-OE-46*, *SNAT-OE-50*, *ASMT-OE-21* and *ASMT-OE-22* were used as overexpressed transgenic plants, which were respectively renamed as *SNAT-OE-1*, *SNAT-OE-2*, *ASMT-OE-1* and *ASMT-OE-2.*
